# Physiologically based pharmacokinetic model of renally cleared antibacterial drugs in Chinese renal impairment patients

**DOI:** 10.1002/bdd.2258

**Published:** 2021-01-12

**Authors:** Cheng Cui, Xiaobei Li, Hao Liang, Zhe Hou, Siqi Tu, Zhongqi Dong, Xueting Yao, Miao Zhang, Xuan Zhang, Haiyan Li, Xiaocong Zuo, Dongyang Liu

**Affiliations:** ^1^ Drug Clinical Trial Center Peking University Third Hospital Beijing People’s Republic of China; ^2^ Institute of Medical Innovation Peking University Third Hospital Beijing People’s Republic of China; ^3^ School of Pharmaceutical Sciences Peking University Beijing People’s Republic of China; ^4^ Janssen China R&D Center Shanghai People’s Republic of China; ^5^ Department of Cardiology Peking University Third Hospital Beijing People’s Republic of China; ^6^ Center of Clinical Pharmacology Third Xiangya Hospital Central South University Changsha People’s Republic of China

**Keywords:** Chinese renal impairment patients, physiologically based pharmacokinetic model, predictive performance, renally cleared antibacterial drugs

## Abstract

To preliminarily develop physiologically based population models for Chinese renal impairment patients and to evaluate the prediction performance of new population models by renally cleared antibacterial drugs. First, demographic data and physiological parameters of Chinese renal impairment patients were collected, and then the coefficients of the relative demographic and physiological equation were recalibrated to construct the new population models. Second, drug‐independent parameters of ceftazidime, cefodizime, vancomycin, and cefuroxime were collected and verified by Chinese healthy volunteers, Caucasian healthy volunteers, and Caucasian renal impairment population models built in Simcyp. Finally, the newly developed population models were applied to predict the plasma concentration of four antibacterial drugs in Chinese renal impairment patients. The new physiologically based pharmacokinetic (PBPK) population models can predict the main pharmacokinetic parameters, including area under the plasma concentration–time curve extrapolated to infinity (AUC_inf_), renal clearance (CL_r_), and peak concentration (*C*_max_), of ceftazidime, cefodizime, vancomycin, and cefuroxime following intravenous administrations with less than twofold error in mild, moderate, and severe Chinese renal impairment patients. The accuracy and precision of the predictions were improved compared with the Chinese healthy volunteers and Caucasian renal impairment population models. The PBPK population models were preliminarily developed and the first‐step validation results of four antibacterial drugs following intravenous administration showed acceptable accuracy and precision. The population models still need more systematic validation by using more drugs and scenarios in future studies to support their applications on dosage recommendation for Chinese renal impairment patients.

## Graphical abstract

The PBPK population models of Chinese renal impaired patients were preliminarily developed by employing demographic and physiological parameters of Chinese patients and results of first steps towards verification by four antibacterial drugs following IV administration showed acceptable accuracy and precision in Chinese patients with mild, moderate, and severe renal impairment.

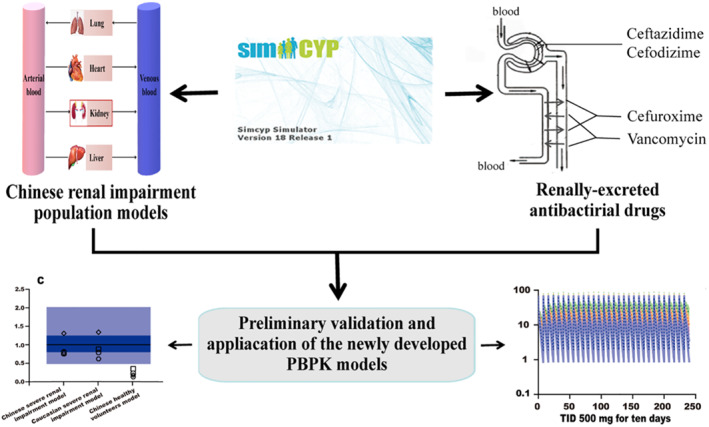

## 1 INTRODUCTION

Antibacterial drugs that are used for the prevention and treatment of clinical infectious diseases are one kind of the most used drugs in the world. According to a national report, up to 834,600 discharged patients multiplied by an average number of hospitalization on average admitted to 166 hospitals with 36.9% average utilization rate of antibacterial drugs, indicating that antibacterial drugs are widely used in clinical practice in China with high frequency (National Health Commission of the People’s Republic of China, 2019). Most antibacterial drugs are eliminated via renal excretion in the form of prototype and/or metabolites (Xu & Wu, 2017). Thus, renal impairment with the outcome of a gradual loss of kidney function may have a great effect on the exposure of antibacterial drugs, hence affecting their efficacy or safety (You, Zhang, Yang, & lijun, 2016). For example, as vancomycin has a longer half‐life in anephric patients, the dosage reduction is recommended in these patients for safety concerns (US Food & Drug Administration, 2017). Although pharmacokinetic (PK) study in patients with various levels of impaired renal function is recommended by the government of China to provide appropriate dosing recommendations, yet it is challenging to recruit renal impairment patients in clinical trials due to safety risk (National Medical Products Administration, 2012). Consequently, such dedicated PK studies for antibacterial drugs are usually not conducted. It was reported that a dose of 33.6% antibacterial drugs were either unadjusted or adjusted only based on foreign data when applied in Chinese patients with renal impairment (Xu & Wu, 2017). However, it may be risky to directly adopt the dosage recommendation of the Caucasian population due to potential ethnic‐related exposure difference. For example, the half‐life of ceftazidime, which was eliminated by passive filtration, in Caucasian patients with severe renal impairment was 1.5‐fold than that of Chinese patients with severe renal impairment (Zhou et al., 2019). Greater differences in patients may be observed in drugs with complex metabolic pathways and mechanisms.

Physiologically based pharmacokinetic (PBPK) model combines the complex interplay of physiological parameters (demographics, organ size, blood flow, etc.) with drug‐related properties (lipid solubility, enzyme, transporter kinetics, etc.) and thus can serve as a mechanistic approach to quantitatively predict the drug PK in different populations (Jamei et al., 2009). Quantitative prediction of the impact of chronic kidney disease (CKD) on drug disposition has become important for the optimal design of clinical studies in patients. PK profiles of 151 compounds under CKD conditions were successfully predicted using a top‐down PBPK approach (Sayama, Takubo, Komura, Kogayu, & Iwaki, 2014). In another study by Scotcher et al., the role of various factors in renal disposition of digoxin has been delineated through the application of the PBPK models on renal impairment population, which provided a rational dose adjustment in clinical application (Scotcher, Jones, Galetin, & Rostami‐Hodjegan, 2017). Recently, a PBPK model has been used to predict plasma concentration of avibactam, a novel β‐lactamase inhibitor, in Caucasian renal impairment patients, with predicted PK parameters within 1.5‐fold of the observed values (Hsueh et al., 2018). With a deeper and more refined understanding of the mechanisms, PBPK models verified with clinical study in healthy volunteers could be potentially applied to predict PK profiles and to recommend dose adjustment for CKD patients (Zhou et al., 2019). However, most models were developed for the Caucasian population whose physiological variables and PK characteristics were not necessarily the same as Chinese, for instance, the physiological characteristics of the kidneys (i.e., kidney weight, kidney blood flow) and the status of the patients with CKD (Cui et al., 2020; Derose et al., 2013).

Thus, the objective of our study is to preliminarily develop physiologically based population models for Chinese renal impairment patients and to evaluate the prediction performance of new population models by renally cleared small‐molecule antibacterial drugs in Chinese renal impairment patients.

## 2 METHODS

**FIGURE 1:**
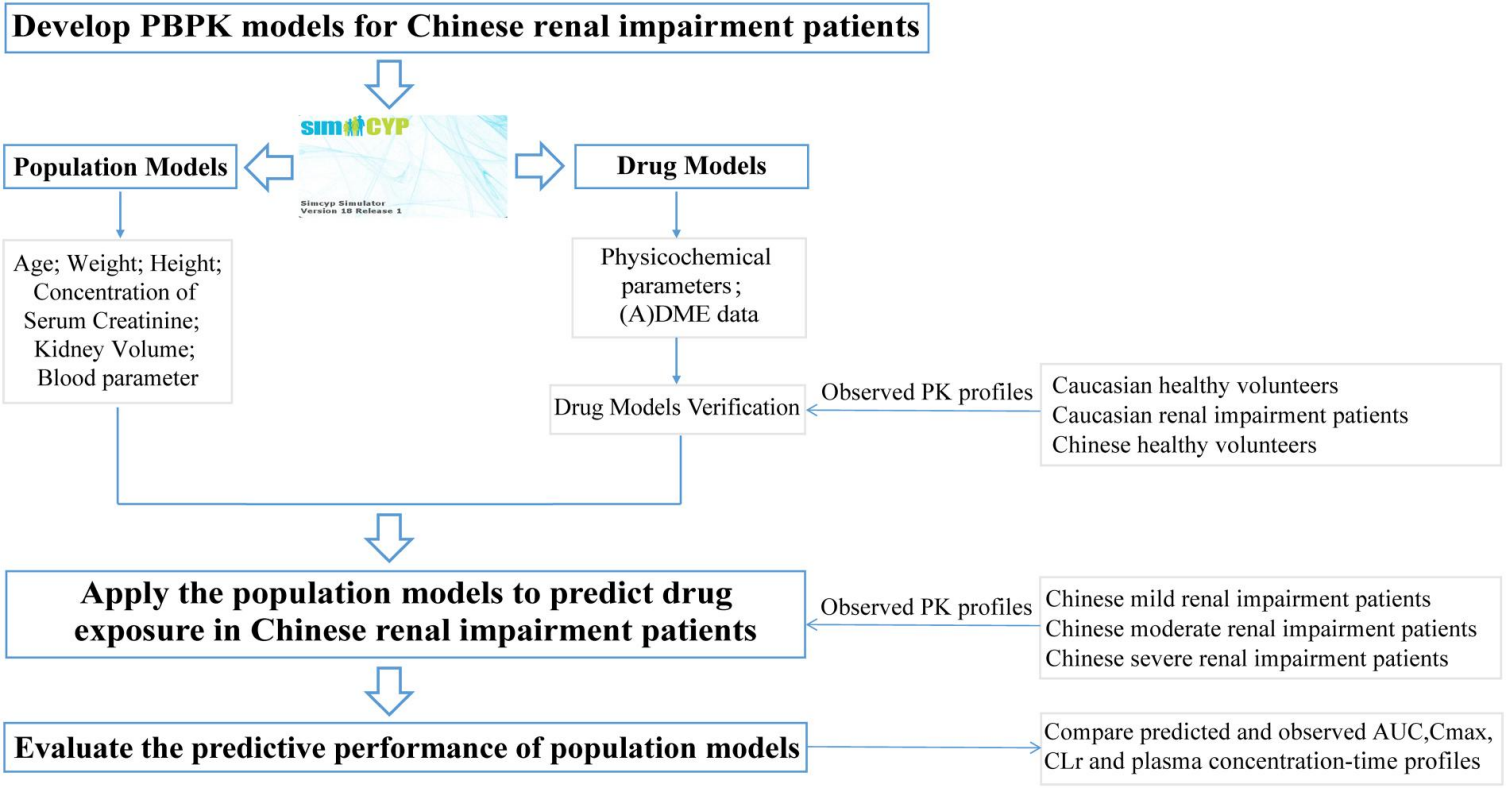
Overall flow diagram of developing, verifying, and applying physiologically based pharmacokinetic models to predict pharmacokinetic characteristics of renally cleared antibacterial drugs in Chinese renal impairment patients with Simcyp

Figure 1 illustrated the approach to develop, verify, and apply PBPK models to predict PK of renally cleared antibacterial drugs in Chinese renal impairment patients using Simcyp (18R1; Certara UK Ltd, Simcyp Division). Briefly, based on the Chinese healthy volunteers' model built in Simcyp, the new PBPK population models were developed and updated with demographic information and serum creatinine concentration values collected from Chinese renal impairment patients. Besides, considering the great effects on drug disposition, the renal volume and blood parameters, which has no sufficient data, were updated by values predefined in Simcyp Caucasian renal impairment population models. Meanwhile, four model drugs were selected and drug‐independent physicochemical properties together with clearance (CL) derived from observed PK data were verified by Caucasian healthy volunteers, Caucasian renal impairment population, and Chinese healthy volunteers' models. After verification, the newly developed population models integrated with drug models were applied to simulate the drug concentration in Chinese renal impairment patients. Finally, the main PK parameters, including the predicted area under the plasma concentration–time curve extrapolated to infinity (AUC_inf_), peak concentration of drug in plasma (*C*_max_), and renal clearance (CL_r_), were compared with the observed data to assess the predictive performance of PBPK population models.

### 2.1 Development of PBPK population models for Chinese renal impairment patients

Glomerular filtration rate (GFR), incorporating serum creatinine concentration along with demographic data, is the most commonly used index of overall kidney function and used to define the various levels of renal impairment in this study (Stevens, Coresh, Greene, & Levey, 2006). The models for Chinese patients with mild (GFR 60–89 ml/min/1.73 m^2^), moderate (GFR 30–59 ml/min/1.73 m^2^), and severe (GFR 15‐29 ml/min/1.73 m^2^) renal impairment were first developed based on the default Chinese healthy volunteers' model in Simcyp by recalibrating age distribution, height, weight parameters, and mean serum creatinine concentration values. The demographic information and serum creatinine in Chinese patients with various renal impairment were collected from the China Kidney Disease Network (CK‐NET) 2015 annual data report and published data (Hu, Li, Yin, & Zuo, 2019; Zhang et al., 2019). The values of hematocrit, α1‐acid glycoprotein, and the relationship of serum albumin and kidney size of Chinese mild renal impairment subjects were adopted from the Chinese healthy volunteers' model, while those parameters of Chinese moderate and severe renal impairment patients were adopted from Caucasian moderate and severe renal impairment population models built in Simcyp. For other parameters, we used the default values in the inbuilt Chinese healthy volunteers' model.

Age and weight were modeled against height using a polynomial equation and an exponential equation, respectively, referring to the Chinese healthy volunteers' model. For serum creatinine, the values were used to calculate GFR with the Cockcroft–Gault equation.

To evaluate if the newly developed population models can adequately describe physiological characteristics in the Chinese renal impairment patients, internal validation was used for height, weight, serum creatinine, and GFR for which 90% confidence interval (CI) of these variables from simulated 4000 subjects (female/total = 0.5; 20 trials, 200 subjects per trial) were compared to observed data.

### 2.2 Development and verification of drug models

The impact of renal impairment on the non‐renal elimination route was not considered and antibacterial drugs that are mainly eliminated by renal excretion were selected in the current study. Based on the drug elimination mechanism and the availability of PK data in Chinese renal impairment patients, four model drugs (i.e., ceftazidime, cefodizime, vancomycin, and cefuroxime) were selected. The literature retrieval strategy was shown in Figure S1. For ceftazidime and vancomycin, the PK data are available in Chinese patients with mild, moderate, and severe renal impairment (Ji et al., 2018; Lin, Wang, & Huang, 1989). For cefodizime and cefuroxime, the PK data are available in Chinese patients with severe renal impairment (Cheng et al., 2010; Li, Song, & Hu, 2000). As the PK data of model drugs in Chinese renal impairment patients were all from clinical studies following intravenous injection (IV) administration, parameters of drug absorption were not involved in our study. For the improvement and comparison of follow‐up research, a full PBPK model was used to describe drug distribution. Depending on drug acid–base properties, the volume of distribution at steady state (*V*_ss_) of ceftazidime, cefodizime, cefuroxime, and vancomycin was initially predicted (Poulin & Theil, 2002; Rodgers & Rowland, 2007). *K*_p_ scalar was determined by fitting the predicted *V*_ss_ to observed clinical *V*_ss_ pharmacokinetic data. For the CL and CL_r_, the values were derived from the PK studies following a single IV administration in Caucasian healthy subjects. Detailed input parameters of the PBPK model for each drug were summarized in Table S1.

The drug models were verified using PK data from Caucasian healthy subjects, Caucasian renal impairment patients, and Chinese healthy subjects. For Caucasian mild renal impairment population model that is not available in Simcyp, Yee et al. established a new mild renal impairment model by using Caucasian healthy volunteers' (18–95 years) model built in Simcyp as a template and increasing concentration of serum creatinine by 1.5‐fold considering that the GFR lower bound cutoff value of mild renal impairment patients is about 1.5‐fold lower than healthy subjects (i.e., 60 vs. 90 ml/min/1.73 m^2^). The other demographic and physiological parameters were kept the same as the Caucasian healthy volunteers' model (Yee et al., 2018).

Trial designs in Simcyp Simulator were set to match the observed clinical studies. The demographic information and study designs for all trials were summarized in Table S2. The simulation was conducted in 10 trials with 10 subjects (effectively used as 100 subjects × 1 trial.) under fasting conditions. The central tendencies were calculated for all virtual subjects. Mean values of simulated and observed data were compared and the rationality of the model was verified when the predicted AUC_inf_, *C*_max_, and CL_r_ were within twofold of the observed values in all studies. The mean absolute prediction error (MAPE) was additionally applied to examine and compare the precision and bias of simulations in PBPK models:

$$MAPE\left(\%\right)=\frac{1}{n}\times \sum_{i}^{n}\frac{\left|\left(C_{\mathrm{Pr}ed,i}-C_{Obs,i}\right)\right|\times 100}{C_{Obs,i}}.$$

### 2.3 Application and predictive performance evaluation of the new PBPK population models

In the combination of the verified drug models, the newly developed Chinese renal impairment population models were then applied to predict plasma concentrations of ceftazidime, cefodizime, vancomycin, and cefuroxime, respectively. The methods of simulation setting and assessment were the same as that of drug parameter verification in the above text.

To further confirm the necessity of the newly developed population models, the Chinese healthy volunteers and Caucasian mild, moderate, and severe renal impairment population models were applied to simulate the same clinical designs of model drugs in Chinese renal impairment patients. Predictive performance among the three models, which was reflected by the ratio of predicted AUC_inf_ versus observed one and MAPE values, were compared.

Besides, the drug ceftazidime was chosen to simulate plasma concentration profiles over time in renal impairment with different levels (mild, moderate, and severe). Three administration regimens (500 mg TID for 10 days; 500 mg BID for 10 days; and 1000 mg QD for 10 days) based on recommended dosage were designed and simulated to demonstrate the application of Chinese renal impaired population models.

## 3 RESULTS

### 3.1 Development of PBPK population models for Chinese renal impairment patients

Chinese renal impairment population models were first developed in Simcyp, for which age, body weight, height, and serum creatinine in Chinese patients with mild, moderate, and severe renal impairment were collected. The age limitation and sex ratio were represented by observational data. The summarized data and equation of system‐dependent parameters of the population models were shown in Tables 1 and 2, and the age distribution of patients with various renal functions was shown in Figure S2.

**TABLE 1:** Summary of physiological data collected from Chinese renal impairment population

Parameters	Mild renal impairment			Moderate renal impairment			Severe renal impairment		
	Males (*n* = 67)	Females (*n* = 21)	Total (*n* = 88)	Males (*n* = 191)	Females (*n* = 85)	Total (*n* = 276)	Males (*n* = 266)	Females (*n* = 162)	Total (*n* = 428)
Age (years)	41.96 ± 13.14	42.76 ± 14.09	42.15 ± 13.29	52.45 ± 13.84	45.73 ± 13.20	50.38 ± 13.97	53.57 ± 15.29	53.18 ± 13.47	53.42 ± 14.62
Height (cm)	167.06 ± 5.58	157.67 ± 4.59	164.82 ± 6.68	167.03 ± 5.44	156.34 ± 4.67	163.74 ± 7.23	167.06 ± 5.40	156.36 ± 4.75	163.01 ± 7.32
Weight (kg)	71.07 ± 9.91	58.38 ± 10.48	68.05 ± 11.38	68.10 ± 9.83	56.22 ± 10.36	64.44 ± 11.39	65.65 ± 10.44	56.11 ± 10.60	62.04 ± 11.47
Serum creatinine (µmol L^−1^)	116.03 ± 16.21	87.86 ± 16.17	109.31 ± 20.13	174.81 ± 45.86	157.25 ± 40.90	169.40 ± 45.06	347.04 ± 96.80	284.41 ± 78.82	323.34 ± 95.30

**TABLE 2:** Summary of the changed system‐dependent parameters in Chinese renal impairment population model

		Mild renal impairment			Moderate renal impairment		Severe renal impairment	
M‐number		67			191		265	
F‐number		21			85		162	
Age range		18–69			22–83		18–83	
Sex ratio (F/Total)		0.22			0.42		0.42	
Age‐BH	M	BH = 169.83 − 0.0891 × Age + 0.0005 × Age^2^			BH = 172.56 − 0.1205 × Age + 0.0003 × Age^2^		BH = 176.04 − 0.2536 × Age + 0.0015 × Age^2^	
	F	BH = 155.95 + 0.1158 × Age − 0.0016 × Age^2^			BH = 168.69−0.4967 × Age + 0.0046 × Age^2^		BH = 155.86 + 0.0672 × Age − 0.001 × Age^2^	
BW‐BH	M	BH = e^2.13+0.0128×BW^			BH = e^2.8+0.0082×BW^		BH = e^1.72+0.0146×BW^	
	F	BH = e^0.93+0.0198×BW^			BH = e^2.89+0.0071×BW^		BH = e^2.10+0.0122×BW^	
Serum creatinine Mean (CV%)	M	Less than 60 years		118 (13)	Less than 50 years	200 (23)	Less than 50 years	411 (23)
		Greater than 60 years		95 (10)	Greater than 50 years	156 (23)	Greater than 50 years	304 (24)
	F	Less than 50 years		93 (14)	Less than 40 years	185 (22)	Less than 30 years	412 (22)
		Greater than 50 years		78 (23)	Greater than 40 years	150 (24)	Greater than 30 years	300 (23)
		－			Greater than 60 years	125 (24)	Greater than 60 years	229 (20)
Hematocrit (%) (CV%)	M	45.3 (9.5)			39.7 (6.5)		33.2 (6.5)	
	F	40.5 (10.9)			36.5 (7.1)		31.3 (7.1)	
AGP (g/L)(CV%)	M	0.638 (23)			0.793 (23)		1.14 (35)	
	F	0.575 (24)			0.715 (24)		1.03 (35)	
HSA (g/L)	M	C0: 50.34 C1: −0.0575 C2: −0.0738			C0: 47.1 C1: −0.0575 C2: −0.0738		C0:43.08 C1: −0.0575 C2: −0.0738	
	F	C0: 49.38 C1: −0.037 C2: 0.1286			C0: 44.9 C1: −0.037 C2: −0.1286		C0:37.8 C1: −0.0575 C2: −0.1286	
Kidney size parameters	Volume	Baseline: 15.4 BW: 2.04 BH: 51.8			Baseline: 8.4 BW: 1.64 BH: 32.8		Baseline: 5.7 BW: 1.04 BH: 29.8	
	Density (g/L)		1050		1050		1050	

Abbreviations: AGP, acid glycoprotein‐α (g/L); BH, body height (cm); BW, body weight (kg); F, female; HSA: human serum albumin; M, male; CV, coefficient of variation.

The 2015 annual data report of CK‐NET included 887,816 hospitalized patients with CKD in 2015, which provided sufficient data for us to determine the sex ratio of Chinese mild and moderate renal impairment patients (Zhang et al., 2019). A cross‐sectional study including 1816 patients, which was from July 2010 to May 2017 in Third Xiangya Hospital of Central South University (Class‐III/Grade‐A hospital) (Hu et al., 2019), provided basic but significant demographic information and serum creatinine concentration source for Chinese mild, moderate, and severe renal impairment patients. As shown in Table 1, the mean age of Chinese mild, moderate, and severe renal impairment patients was 42.15 ± 13.14, 50.38 ± 13.97, and 53.42 ± 14.62 years, respectively. It seems that patients with mild renal impairment have a lower age distribution. The mean height of the three renal levels population was 164.82 ± 6.68, 163.74 ± 7.23, and 163.01 ± 7.32 cm, showing no significant differences (lower <1%) among the three populations. The mean value of weight decreases by 4%–5%, of which the value was 68.05 ± 11.38, 65.20 ± 11.39, and 62.04 ± 11.47 kg as renal function loss. The loss in weight is more significant in males than in females. The mean values of serum creatinine concentration were 109.31 ± 20.13, 169.40 ± 45.06 (about 1.5‐fold higher than mild renal impairment), and 323.34 ± 95.30 µmol L^−1^ (about twofold higher than moderate renal impairment).

**FIGURE 2:**
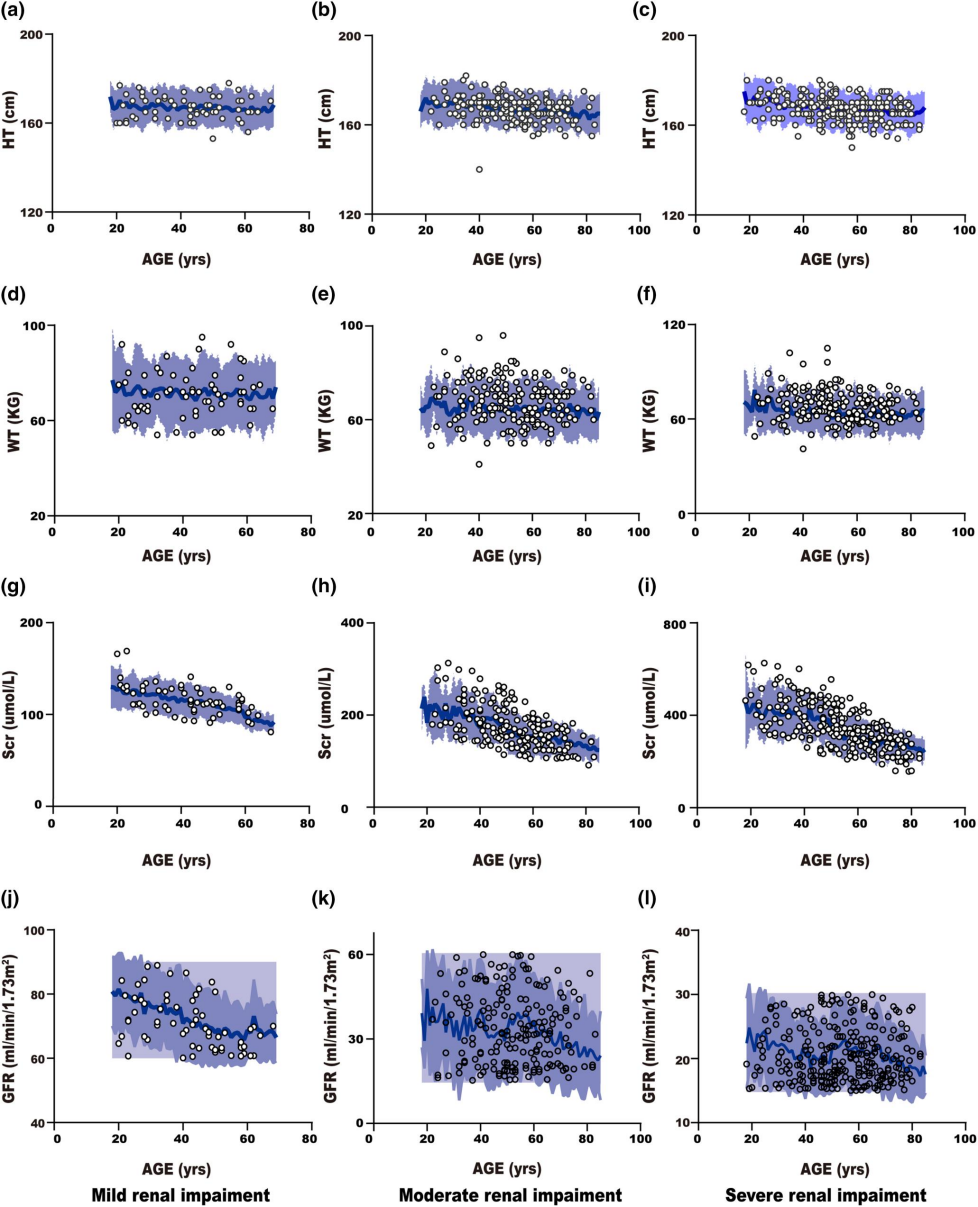
Simulated versus observed body height, body weight, serum creatinine and glomerular filtration rate as a function of age in the Chinese male population with various levels of renal impairment. The blue area represents a 90% confidence interval from 2000 virtual individuals simulated in physiologically based pharmacokinetic population models and the solid blue line represents mean values. Black rings represent observed values from the public data (Hu et al., 2019) study database

Figure 2 showed that most observed height, weight, serum creatinine, and GFR values of Chinese male renal impairment patients were within the 90% CI of the simulated values, and the results were the same as in Chinese female renal impairment patients (Figure S3).

### 3.2 Development and verification of drug models

**FIGURE 3:**
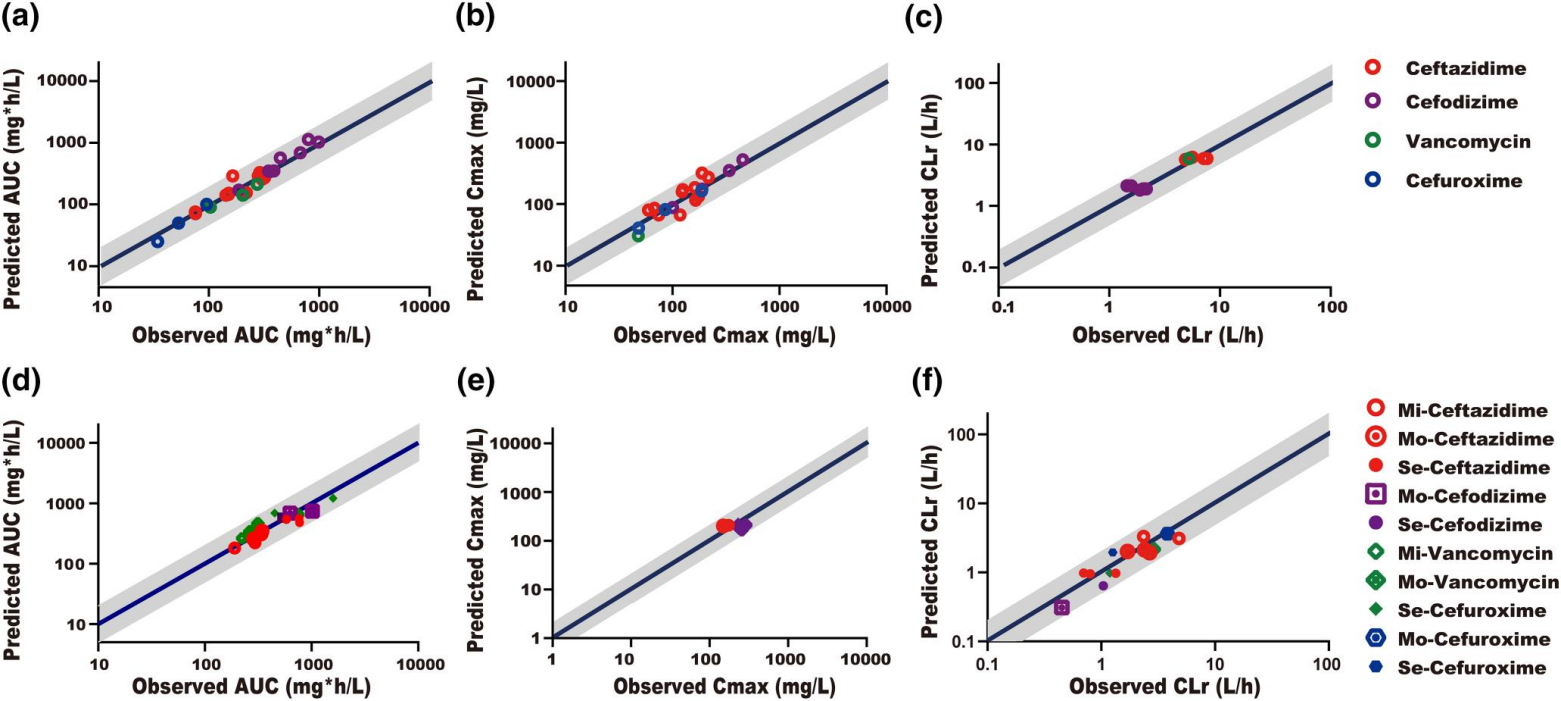
Predicted versus observed (a) AUC_inf_ (b) C_max_, and (c) CLr in Caucasian, Chinese healthy subjects and (d) AUC_inf_ (e) C_max_ (f) CLr in Caucasian renal impairment patients. The gray area represents the twofold error range. The blue solid line represents the identity line. AUC_inf_, the area under the plasma concentration‐time curve extrapolated to infinity; *C*_max_, maximum (peak) concentration of drug in blood plasma; CL_r_, renal clearance; Mi, mild renal function reduction; Mo, moderate renal function reduction; Se, severe renal function reduction

The drug models for ceftazidime, cefodizime, vancomycin, and cefuroxime were developed to predict plasma drug concentration following IV administration. The models were then verified with PK data from Caucasian healthy subjects, Chinese healthy subjects, and Caucasian renal impairment subjects. As shown in Figure 3a–c and Table S3, the predicted AUC_inf_, *C*_max_, and CL_r_ in Caucasian healthy subjects and Chinese healthy subjects for all drugs were within twofold of observed values where 78% of predicted AUC_inf_ and 85% of predicted CLr were within 0.8–1.25‐fold, while 47% of predicted C_max_ were within 0.8–1.25‐fold of the observed values. For Caucasian renal impairment subjects, as was shown in Figure 3d–f and Table S4, the predicted AUC_inf_, C_max_, and CL_r_ were all within twofold of the observed values. Among patients with mild renal impairment, 75% of predicted AUC_inf_ and 100% of predict *C*_max_ (only one for ceftazidime) were within 0.8‐1.25‐fold of the observed value; however, none of the predicted CLr was within 0.8–1.25‐fols of the observed values. As for subjects with moderate renal impairment, 67% of predicted AUC_inf_, 33% of predicted *C*_max_, and 50% of predicted CL_r_ were within 0.8–1.25‐fold of the observed values. For patients with severe renal impairment, 25% of predicted AUC_inf_. 33% of predicted CL_r_, and none of *C*_max_ were within 0.8–1.25‐fold of the observed values. Overall, these results suggested that drug‐dependent parameters were adequately inputted in our drug models.

### 3.3 Application and predictive performance evaluation of the new PBPK population models

**FIGURE 4:**
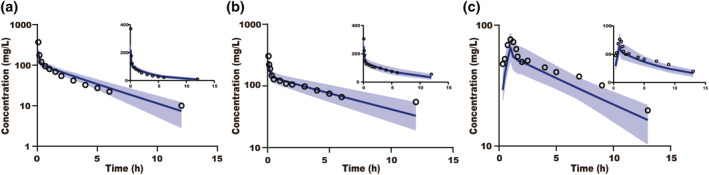
Simulated and observed plasma concentration–time profiles for ceftazidime in Chinese patients with mild renal impairment (a) and glomerular filtration rate 15–60 ml/min/1.73 m^2^ (b) and cefuroxime in Chinese patients with severe renal impairment (c). The blue solid line represents the mean simulated concentration and the blue area represents the 5th and 95th percentiles of the simulated concentration. Black rings are the mean of the observed data

As was presented in Table S5, the predicted AUC_inf_ for model drugs following single IV administration in Chinese renal impairment patients were within twofold of the observed values. Specifically, 50%, 100%, and 40% of prediction on AUC_inf_ were within 0.8–1.25‐fold of the observed values in mild, moderate, and severe renal impairment patients, respectively. Only the observed CL_r_ of cefodizime and observed *C*_max_ of cefuroxime were available in Chinese severe renal impairment patients, which were in the range oftwofold and 0.8–1.25‐fold of the predicted values, respectively. Also, all predicted plasma concentration profiles were in good agreement with observed data that were available in the public domain and illustrated in Figure 4.

**FIGURE 5:**
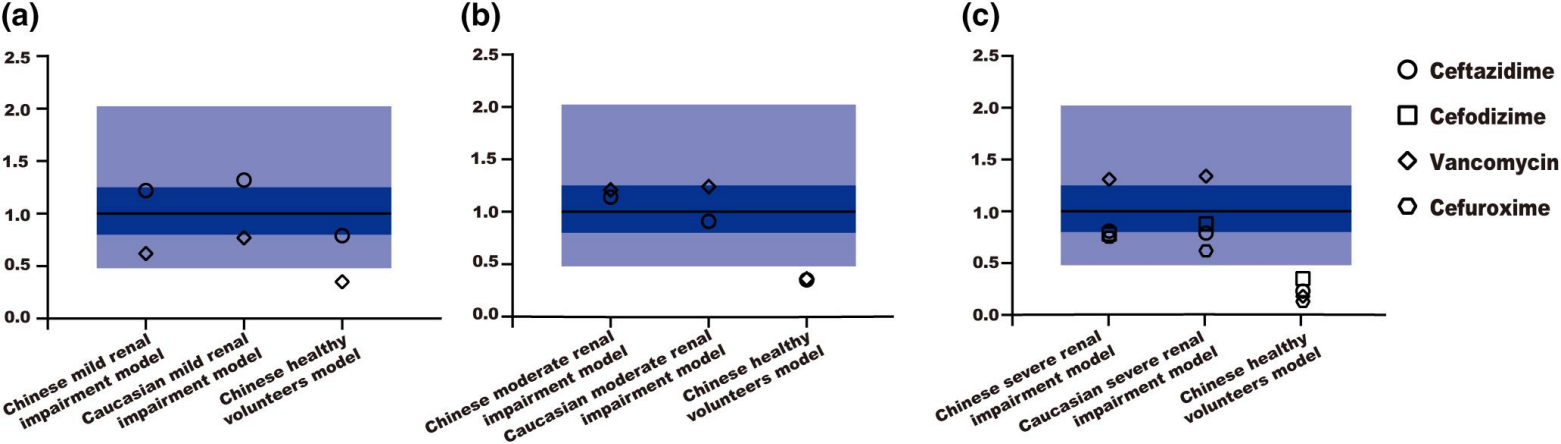
Comparison of AUC_inf_ prediction performance of different population models. The solid black line represents the identity line; Dark blue and light blue areas show 0.8–1.25‐fold error range and 0.5–2‐fold error range, respectively. AUC_inf_, area under the plasma concentration–time curve extrapolated to infinity

As shown in Table S6 and Figure 5, when compared with the Chinese healthy volunteers and Caucasian impaired population models, the value of MAPE significantly decreased and the predicted AUC_inf_ simulated by Chinese renal impairment population models were much closer to the observed data, indicating improved precision and accuracy by our new population models.

**FIGURE 6:**
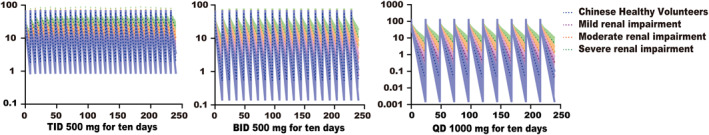
Simulated plasma concentration‐time profiles of ceftazidime after multiple doses using the newly developed Chinese renal impairment models

Figure 6 showed the ceftazidime plasma concentration–time curves under the scenarios of three administration regimens simulated by the newly developed Chinese renal impairment population models. The flexibility of PBPK, combined with the pharmacodynamic characteristics of the drug, can provide evidence and support dose selection for various levels of renal impairment population in clinical practice.

## 4 DISCUSSION

Antibacterial drugs are frequently used in China. Although many of them are predominantly eliminated via renal and may have altered drug exposure in Chinese renal impairment patients, dose adjustment is usually not conducted in these patients due to a lack of corresponding PK data (Xu & Wu, 2017). To fill this gap, we explore the possibility to use PBPK models to predict the antibacterial drug concentrations in Chinese renal impairment patients. First, demographic data and the serum creatinine concentration values of Chinese renal impairment patients were collected to recalibrate the demographic and physiological equations. The verified drug parameters combined with the primarily developed population models were applied to predict drug concentration in Chinese patients with various levels of renal impairment. Our study suggested that the precision and accuracy of the new population models for the prediction of renally cleared antibacterial PK characteristics were improved significantly.

Renal impairment is usually associated with various physiological changes. In Caucasian renal impairment patients, body weight has a nonlinear change that it was stable or slightly increased until cystatin C‐based eGFR fell below 35 ml/min/1.73 m^2^; every 10 ml/min/1.73 m^2^ decline in GFR was associated with a mean 2.3 kg (95% CI, 2.1–2.4 kg) weight decline (Ku et al., 2018). Besides, the hematocrit decreased progressively until the estimated creatinine clearance (CL_cr_) is below 60 ml/min in men and 40 ml/min in women (Hsu, Bates, Kuperman, & Curhan, 2001). It was also reported that there was a significant decrease in the concentration of serum albumin for moderate and severe renal impairment patients (Amin, El‐Sayed, & Leheta, 2016). In contrast, α1‐acid glycoprotein showed a significant increase (*p* < 0.05) (Romao et al., 2006) in patients with severe renal impairment while no significant differences were found in mild and moderate renal impairment patients. Moreover, the kidney volume was shown to decrease with the progress of CKDs (Vegar Zubovic, Kristic, & Sefic Pasic, 2016).

In Chinese renal impairment patients aged 18–85, nearly 50% of the patients were older than 60 years old (Wang et al. 2019a). Among them, the number of men was generally higher than that of women by about 1.5‐fold (Tables 1 and 2). Like Caucasian renal impairment patients, the body weights of Chinese renal impairment patients remain stable with GFR > 30 ml/min, and then decrease with the reduction of renal function (Wang et al. 2019b).

Kidney volume, hematocrit, the concentration of serum albumin, and α1‐acid glycoprotein were also affected by various renal functions, but have not been reported in Chinese renal impairment patients so far. For kidney size, it was reported that kidney volume decreases significantly in Chinese moderate and severe renal impaired patients, while there were no significant differences between Chinese patients with mild renal impairment and Chinese healthy subjects (Shen et al., 2013, Tian 2015). For hematocrit, human serum albumin, and α1‐acid glycoprotein, due to the lack of data, we hypothesized that the values of three blood parameters changed as it did in patients with Caucasian renal impairment population. All of them were changed significantly in severe renal impairment patients, while no significant differences in mild renal impairment patients were found compared with healthy subjects (Amin et al., 2016; Hsu et al., 2001; Romao et al., 2006). Both the hematocrit and concentration of human serum albumin declined significantly in patients with moderate renal impairment patients, while little evidence of changes was found for α1‐acid glycoprotein, in consistent with the inbuilt parameter value in Simcyp (Amin et al., 2016; Hsu et al., 2001; Romao et al., 2006). Thus, when the population model was developed for Chinese patients with moderate and severe impairment, these system‐dependent parameters are directly adopted from Caucasian patients based on the assumption that the alteration of these parameters is to the same extent between Chinese and Caucasian renal impairment patients.

In this study, the antibacterial drugs ceftazidime, cefodizime, vancomycin, and cefuroxime, which are predominantly cleared via renal elimination, were used as model drugs to explore the predictive performance of developed population models. Compared with the observed data of four model drugs in Chinese patients with renal impairment, all predicted PK parameters (i.e., AUC_inf_, *C*_max,_, and CL_r_) were within twofold of the observed data. Besides, the MAPE was used to evaluate the predictive ability and systematic prediction biases. The Chinese healthy volunteers model generally underestimated the exposure of model drugs in patients with various renal impairments. Particularly, the predicted values of the model in patients with moderate and severe renal impairment were lower than 50% of observed data, indicating that renal function loss exerts great effect on drug clearance. Although the predicted AUC_inf_ values in Caucasian renal impairment population models were within twofold of the observed value as that in the Chinese renal impairment population models, the Chinese renal impairment population models exhibited higher accuracy and precision. Among these four drugs, ceftazidime and cefodizime, which were primarily excreted by glomerular filtration, showed better predictive performances. For vancomycin, it was reported that 25% of the drugs had (Table S1) renal tubular reabsorption (Kusama et al., 1998), whereas cefuroxime was suggested to be a substrate of multidrug resistance‐associated protein 4 (MRP4) (Akanuma et al., 2011) with 28% (Table S1) of drugs being actively secreted. The evidence implies the importance to incorporate renal transporters (e.g., transport activity data) in the model to better predict the PK characteristics of drugs that have significant active secretion or reabsorption.

Though the predicted PK profiles and parameters for these different drugs were generally in agreement with the observed data, our study had some limitations. First, due to the lack of hematocrit, serum albumin, and kidney volume data in the Chinese population, these data were assumed to be the same as Caucasian renal impairment patients. This assumption requires further verification and confirmation even though our model presented reasonable prediction on the PK of all four drugs in Chinese patients with mild, moderate, and severe renal impairment. Second, for drugs with high degree of active transport, the current model is insufficient to accurately predict their PK profile and parameters, and further development of mechanistic kidney models based on Chinese data is needed (Scotcher, Jones, Posada, Galetin, & Rostami‐Hodjegan, 2016). Lastly, apart from the well‐recognized effects of kidney disease on renal drug clearance, there is a growing body of evidence demonstrating that renal failure may influence hepatic drug metabolism either by inducing or suppressing hepatic enzymes or by its effects on protein binding, hepatic blood flow, and accumulation of metabolites (Follman & Morris, 2018; Rowland Yeo, Aarabi, Jamei, & Rostami‐Hodjegan, 2011; Zhao et al., 2012). In our study, though a minor yet substantial (>25%) route of cefodizime elimination is non‐renal, the predicted results were within 1.25‐fold of the observed values. Further studies are needed to consider changes in non‐renal clearance by including more drugs with larger non‐renal clear proportion for model optimization and validation.

## 5 CONCLUSION

The PBPK population models by employing demographic and physiological parameters of Chinese patients with renal impairment were preliminarily developed and the results of first step toward verification by four antibacterial drugs following IV administration showed acceptable accuracy and precision in Chinese patients with mild, moderate, and severe renal impairment. In future studies, the model should be systematically validated using more drugs with various elimination mechanisms and diverse administrations to support its application in dosage recommendation in Chinese renal impairment patients even in the absence of a dedicated PK study.

## CONFLICT OF INTEREST

The authors have no conflict of interest to disclose.

## Supporting information

Supplementary Material

Supplementary Material
