# Supplementary material for: Physiologically based pharmacokinetic model of renally cleared antibacterial drugs in Chinese renal impairment patients
Source: Biopharm Drug Dispos. 2021 Jan 12;42(1):24–34. doi: 10.1002/bdd.2258 (PMC7898311; doi:10.1002/bdd.2258)
Supplement: Supplementary file 1 — Supplementary Material [file BDD-42-24-s001.docx]

Physiologically Based Pharmacokinetic Model of Renally Cleared Antibacterial Drugs in Chinese Renal Impairment Patients

Cheng Cui^1,2 ‡^, Xiaobei Li^1,3, ‡^, Hao Liang^1,2^, Zhe Hou^1,2^, Siqi Tu^1,2^, Zhongqi Dong^4^, Xueting Yao^1,2^, Miao Zhang^1,2^, Xuan Zhang^3^, Haiyan Li^1,2,5^, Xiaocong Zuo^6, *^, Dongyang Liu^1,2, *^

*^1^ Drug Clinical Trial Center, Peking University Third Hospital, Beijing, 100191, China*

*^2^ Institute of Medical Innovation, Peking University Third Hospital, Beijing 100191, China.*

*^3^ School of Pharmaceutical Sciences, Peking University, Beijing, 100191, China*

*^4^ Janssen China R&D Center, Shanghai, 200233, China*

*^5^ Department of Cardiology, Peking University Third Hospital, Beijing, 100191, China.*

*^6^ Center of Clinical Pharmacology, Third Xiangya Hospital, Central South University, Changsha, 410013, China*

^‡^ Cheng Cui and Xiaobei Li contributed equally to this work.

**Correspondence:*

*Dongyang Liu, Drug Clinical Trial Center, Peking University Third Hospital, Beijing, 100191, China. (liudongyang@vip.sina. com)*

*Xiaocong Zuo, Center of Clinical Pharmacology, Third Xiangya Hospital, Central South University, Changsha, 410013, China (zuoxc08@126.com)*

**
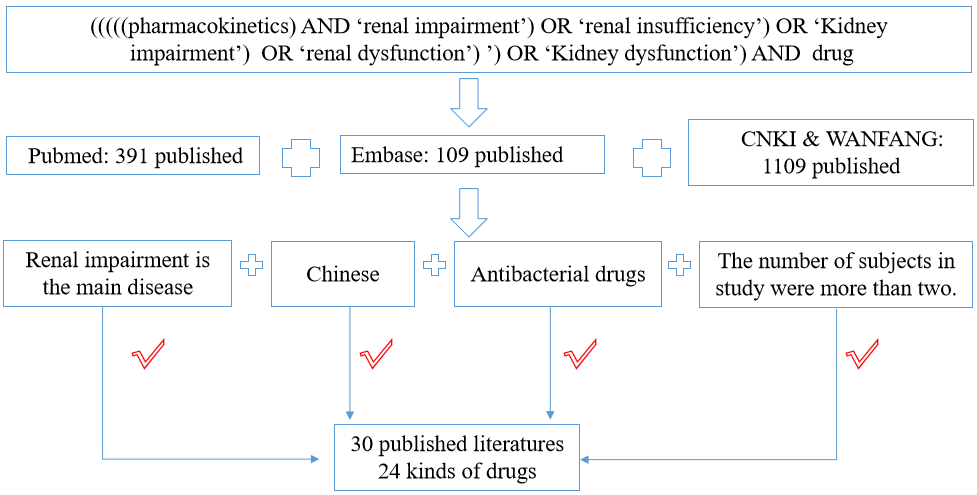
**


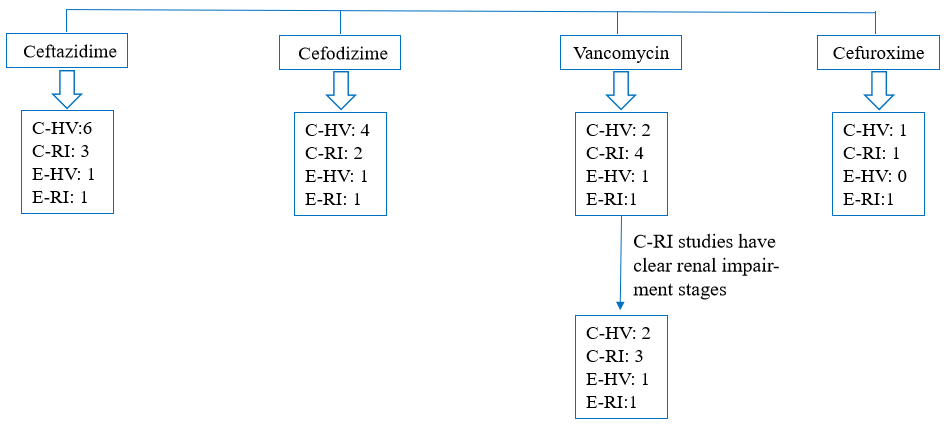

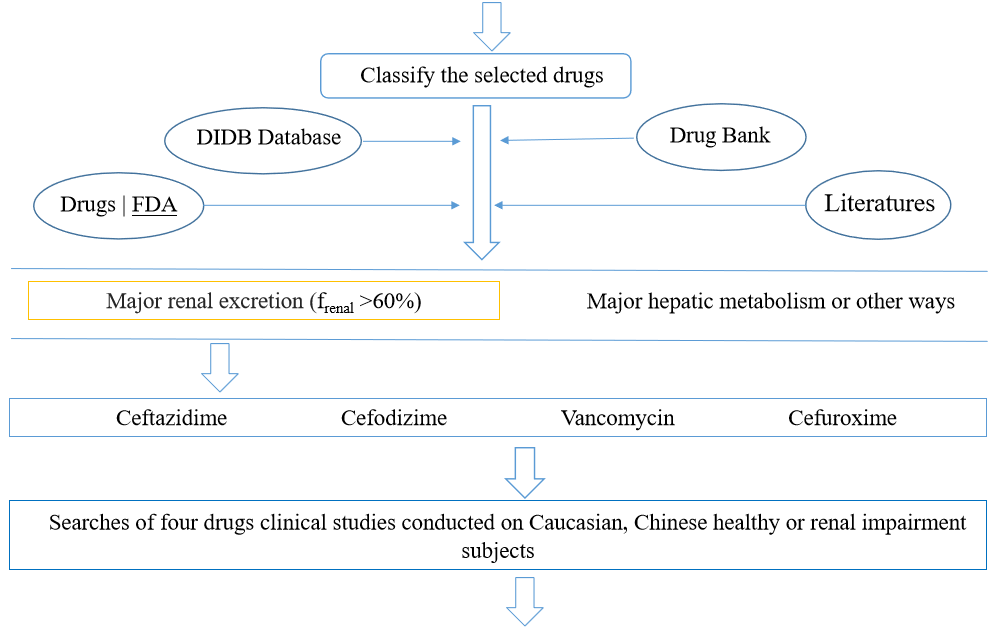


**Supplemental Figure S1.** Drug and literature retrieval strategy. C-HV, Caucasian healthy volunteers; C-RI, Caucasian renal impairment patients; E-HV, Chinese healthy volunteers; E-RI, Chinese renal impairment patients.


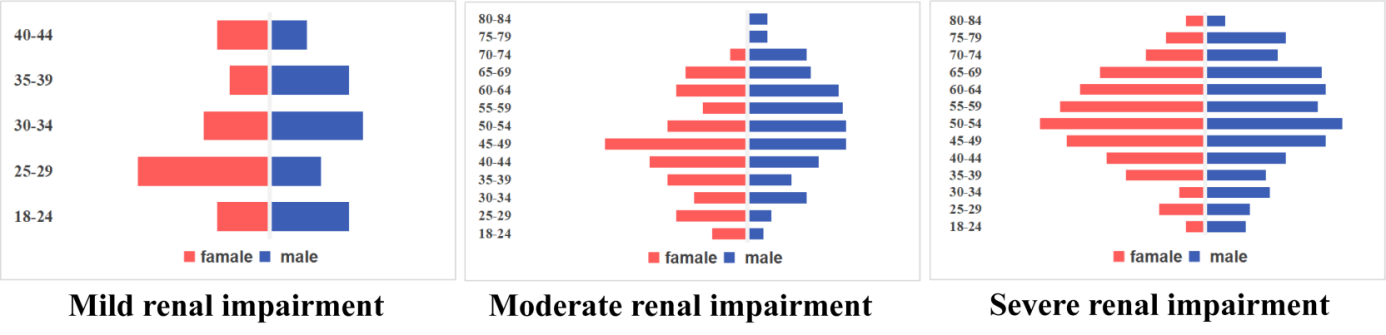
**Supplemental Figure S2. The age distribution of the Chinese renal impairment population.**

**
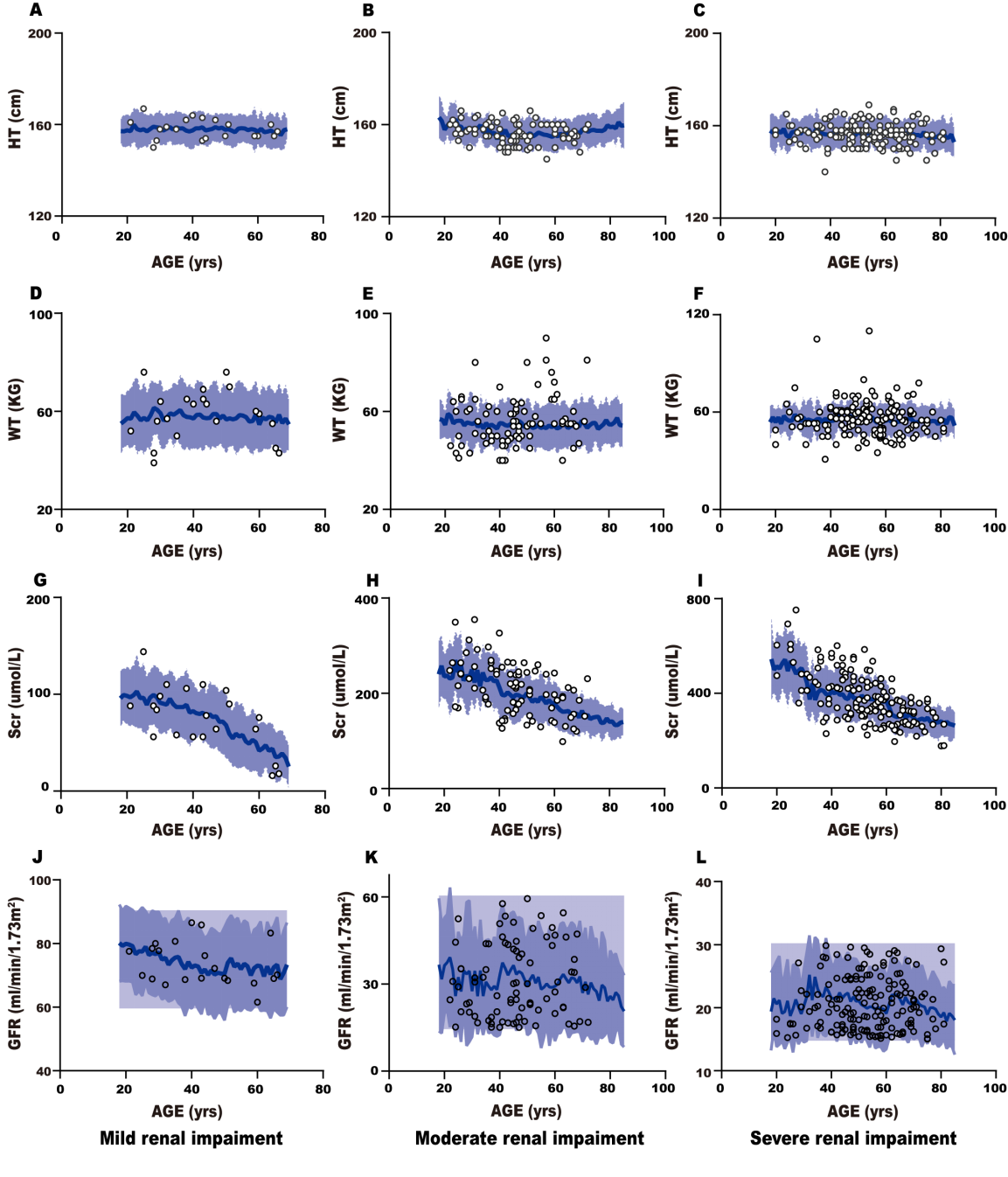
**

**Supplemental Figure S3.** Simulated vs observed body height, body weight, serum creatinine and glomerular filtration rate (GFR) as a function of age in the Chinese female population with various levels of renal impairment. The blue area represents a 90% confidence interval from 2000 virtual individuals simulated in PBPK population models and the solid blue line represents mean values. Black rings represent observed values from the public data (Hu et al. 2019) study database.

**Supplemental Table S1 Summary of input parameters for input specific parameters for ceftazidime, cefodizime, vancomycin and cefuroxime**

| **Parameter** | **Ceftazidime** | **Cefodizime** | **Vancomycin** | **Cefuroxime** |
| --- | --- | --- | --- | --- |
| **Molecular weight (g/mol) ^a^** | 546.58 | 584.67 | 1449.26 | 424.388 |
| **log P^a^** | −2.65 | 0.524 | -3.75 ^[6]^ | 1.757 |
| **Compound type** | Acid | Acid | Amph | Acid |
| **pKa1^a^** | 2.40 | 1.7 | 2.18 ^[6]^ | 2.5 ^[7]^ |
| **pKa2^a^** | 4.26 | 2.47 | 7.75 ^[6]^ | — |
| **fu** | 0.9 ^[1]^ | 0.185 | 0.672 ^[6]^ | 0.6^[7]^ |
| **Distribution model** | Full PBPK model | | | |
| **V_ss_ (L/kg)** | Method 2 | Method 2 | Method 1 | Method 2 |
| **K_p_ scalar^b^** | 1.5 | 1 | 1 | 1 |
| **CL_r_ (L/h)** | 6 ^[1]^ | 1.86^[2,3,4,5]^ | 6 ^[6]^ | 10.4 ^[8]^ |
| **CL_nr_ (L/h)** | 0.9 ^[1]^ | 1.08^[2,3,4,5]^ | — | — |

Amph ampholyte; CL_r_ renal clearance; CLnr non-renal clearance; CXM, cefuroxime; fu fraction of unbound drug in plasma; logP log of the octanol-water partition coefficient for the compound; pKa acid dissociation constant; V_ss_ apparent volume of distribution at steady-state.

^a^Unless otherwise specified, values were collected from online resources such as Scifinder (https://sso.cas.org/ as/S7jUT/resume/ as/authorization.ping/), drug bank (https://www.drug bank.ca/) and pubchem (https://pubchem. ncbi.nlm.nih.gov/); ^b^ Kp scalar was adjusted to match clinical observations.

| [1] US FOOD & DRUG ADMINISTRATION (2015) | [2] (Bryskier et al. 1990) | [3] (Bryskier et al. 1990) |
| --- | --- | --- |
| [4] (Lenfant et al. 1995) | [5] (Korting et al. 1987) | [6] (Abduljalil et al. 2019) |
| [7] (Dallmann et al. 2017) | [8] (Schwameis et al. 2017) |  |

**Supplemental Table S2 Specific experimental design for four drugs simulated in the study**

| **Drug** | **Condition** | **Population** | **Source** | **Trial Design** |
| --- | --- | --- | --- | --- |
| **Ceftazidime** | Healthy | Caucasian | (Harding and Harper 1983) | A Single iv bolus of 500mg, 1000mg, 2000mg, iv infusion of 1000mg ,2000mg over 0.33 h; And 2 g doses were given by 10 min infusion at 8-hourly intervals over ten days; Aged 20-49 years; 0% female. |
|  |  |  | (Drusano et al. 1984) | A Single iv infusion of 2000mg over 30 min; Aged 18-35 years; 0% female. |
|  |  |  | (Harding et al. 1981) | A Single iv bolus of 500mg, 1000mg; Aged 34 years; 0% female. |
|  |  |  | (Bulitta et al. 2010) | A Single iv bolus of 2000mg over 5 min; Aged 19-33 years; 43% female. |
|  |  |  | (Kalman et al. 1992) | A Single iv infusion of 2000mg over 30 min; Aged 29 ± 5 years; 0% female. |
|  |  |  | (Ljungberg and Nilsson-Ehle 1984) | Twice daily for at least 7 days as an intravenous bolus injection over 3 min; Aged 23-31 years; 0% female. |
|  |  | Chinese | (Guangquan. et al. 1994) |  |
|  | Renal  Impairment | Caucasian | (Leroy et al. 1984) | A Single iv bolus of 15mg/kg over 3 min after an overnight fast; Aged 26-74 years. |
|  |  |  | (van Dalen et al. 1986) | A Single iv bolus of 1000mg over 2 min; Aged 34-88 years; 30% female. |
|  |  |  | (Welage et al. 1984) | A Single iv bolus of 1000mg over 2-3 min after fasting for 8h; Aged 27-91years; 14% female. |
|  |  | Chinese | (LinMS. et al.) | A Single or multiple iv bolus of 2000mg; Aged 21-75 years; 36% female. |
| **Cefodizime** | Healthy | Caucasian | (Bryskier et al. 1990) | A Single iv infusion of 1000mg, 2000mg over 3 min after an overnight fast for 10 h; Aged 20-28 years; 50% female. |
|  |  |  | (Bryskier et al. 1990) | A Single iv infusion of 1000mg over 3 min after an overnight fast for 10 h; Aged 24-34 years; 0% female. |
|  |  |  | (Lenfant et al. 1995) | A Single iv infusion of 500mg, 1000mg, 2000mg, 3000mg over 5 min after an overnight fasting; Aged18-40 years; 0% female. |
|  |  |  | (Korting et al. 1987) | A Single iv infusion of 1000mg over 3 min; Aged 24-31years; 0% female. |
|  | Renal  Impairment | Caucasian | (Loffreda et al. 1999) | A Single iv bolus of 1000mg over 5 min after fasting for 12h; Aged 18-70 years. |
|  |  |  | (el Guinaidy et al. 1991) | A Single iv bolus of 1000mg after an overnight fast;Aged 19-62 years; 0% female. |
|  |  | Chinese | (Li et al. 2000) | A Single iv bolus of 16.2mg/kg; 42% female. |
| **Vancomycin** | Healthy | Caucasian | (Blouin et al. 1982) | A Single iv infusion of 23.4 ± 1.5 mg/kg over 40 min; Aged 25-30 years; 0% female. |
|  |  |  | (Krogstad et al. 1980) | A Single iv infusion of 500 mg over 30 min; Aged 34-40 years; 0% female. |
|  |  | Chinese | (Jinyu et al. 2003) | A Single iv infusion of 1000 mg over 100 min; Aged 23±2.7 years; 0% female. |
|  | Renal  Impairment | Caucasian | (Hurst et al. 1990) | A Single iv infusion of 15mg/kg over 60 min; Aged 42-67 years; 25% female. |
|  |  |  | (Matzke et al. 1984) | A Single iv infusion of 15.34mg/kg to patients with GFR 60-90 ml/min, 20mg/kg to patients with GFR 30-60 ml/min and 17.5mg/kg to patients with GFR <30 ml/min over 60 min;Patients with GFR 60-90 ml/min were 30-68 years, GFR 30-60 ml/min were 17-85 years and patients with GFR <30 ml/min were 47-74 years; 38% female. |
|  |  |  | (Rodvold et al. 1988) | A Single iv infusion 11.6 mg/kg to patients with GFR 30-60 ml/min and 11.0mg/kg to patients with GFR <30 ml/min over 60 min; Patients were 35-87 years; 30% female. |
|  |  | Chinese | (Ji et al. 2018) | A Single iv infusion of 1000mg over 60 min; Aged 42-95 years;34% female. |
| **Cefuroxime** | Healthy | Caucasian | (Foord 1976) | A Single iv bolus of 250mg, 500mg, 1000mg over 3min after an overnight fasting; Aged 19-57 years; 0% female. |
|  | Renal  Impairment | Caucasian | (van Dalen et al. 1979) | A Single iv bolus of 1000 mg to subjects with 30-60 ml/min and 1500mg to patients with GFR <30 ml/min over 2 min; Ages 14-72 years; |
|  |  | Chinese | (Cheng et al. 2010) | A Single iv infusion of 1000 mg to patients GFR <30 ml/min over 1 h; Patients were 34-55 years; 30% female. |

**Supplemental Table S3 Predicted versus observed PK parameters in Caucasian and Chinese healthy population**

| **Drug** | **Population** | **Age**  **(years)** | **Dose(mg)**  **Route** | **AUC_inf_ (mg·h /L) Mean (SD)** | | | **C_max (_mg/L) Mean (SD)** | | | **CLr (L/h) Mean (SD)** | | | **MAPE**  **(%)** |
| --- | --- | --- | --- | --- | --- | --- | --- | --- | --- | --- | --- | --- | --- |
|  |  |  |  | **Obs** | **Pre** | **P/O** | **Obs** | **Pre** | **P/O** | **Obs** | **Pre** | **P/O** |  |
| **Ceftazidime** | Caucasian | 20-49 ^[1]^ | 500  iv.bolus | 71.9  (1.8) | 71.0  (10.6) | 0.99 | 57.6  (3.8) | 79.8  (7.8) | 1.39 | NA |  |  | 19.78 |
|  |  |  | 1000  iv.bolus | 135.8  (9.5) | 142.0  (21.1) | 1.05 | 119.1  (9.50) | 159.5  (15.52) | 1.34 | NA |  |  | 16.2 |
|  |  |  | 1000  inf | 143.2  (4.5) | 145.4  (21.3) | 1.02 | 72.1  (3.60) | 66.7  (6.33) | 0.94 | NA |  |  | 19.18 |
|  |  |  | 2000  iv.bolus | 279.4  (8.5) | 298.86  (42.8) | 1.07 | 182.8  (16.3) | 319.0  (31.1) | 1.75 | NA |  |  | 35.3 |
|  |  |  | 2000  inf | 266.0  (8.4) | 290.8  (42.6) | 1.09 | 170.0  (6.8) | 133.24  (12.5) | 0.78 | NA |  |  | 31.3 |
|  |  |  | 2000-inf  mutiple | 274.7  (9.7) | 327.8  (54.0) | 1.19 | 156.7  (10.2) | 185.1  (16.4) | 1.18 | NA |  |  | 30.74 |
|  |  | 18-35 ^[2]^ | 2000  inf | 287  (56) | 256  (41) | 0.89 | 159.3  (55.2) | 116.1  (12.4) | 0.73 | 5.1  (0.7) | 6.2  (1.0) | 1.22 | 22.8 |
|  |  | 34 ^[3]^ | 500  iv.bolus | 71.4 | 75.6 | 1.05 | 65.8 | 85.65 | 1.30 | 6.9 | 5.9 | 0.86 | 34.48 |
|  |  |  | 1000  iv.bolus | 143.8 | 151.2 | 1.05 | 121.0 | 171.3 | 1.42 | 6.48 | 5.90 | 0.91 | 27.83 |
|  |  | 19-33 ^[4]^ | 2000  iv.bolus | 303.5 | 270.8 | 0.89 | 210 | 272 | 1.30 | NA |  |  | 10.78 |
|  |  | 29 ^[5]^ | 2000  inf | 156.3  (51.1) | 289.7  (42.0) | 1.85 | NA |  |  | NA |  |  | 146.17 |
|  |  | 23-31 ^[6]^ | 2000-inf  mutiple | 276.7  (9.8) | 285.8  (442) | 1.03 | NA |  |  | 4.9  (0.25) | 6.01  (1.03) | 1.23 | 4.37 |
|  | Chinese | 33 ^[7]^ | 1000  inf | 207.8  (61.7) | 155.2  (22.3) | 0.75 | 114.3  (33.5) | 66.7  (7.1) | 0.58 | 4.42  (1.00) | 5.72  (0.99) | 1.29 | 4.94 |
| **Cefodizime** | Caucasian | 20-28 ^[8]^ | 1000  inf | 422  (43) | 565  (65.34) | 1.33 | NA | | | 1.31  (0.18) | 2.12  (0.40) | 1.62 | 58.01 |
|  |  |  | 2000  inf | 757  (39) | 1130  (130) | 1.49 | NA | | | 1.43  (0.08) | 2.12  (0.40) | 1.24 | 52.46 |
|  |  | 24-34 ^[9]^ | 1000  inf | 368.2  (25.7) | 349.3  (51.4) | 0.95 | NA | | | 1.71  (0.15) | 1.82  (0.29) | 1.06 | 16.14 |
|  |  | 18-40 ^[10]^ | 500  inf | 177  (33) | 171  (27) | 0.97 | 97  (21) | 88  (10) | 0.91 | 1.94  (0.42) | 1.89  (0.29) | 0.97 | 38.45 |
|  |  |  | 1000  inf | 341  (80) | 342  (53) | 1.00 | 183.0  (48.0) | 175.2  (19.1) | 0.95 | 1.83  (0.28) | 1.89  (0.29) | 0.97 | 31.92 |
|  |  |  | 2000  inf | 640  (73) | 685  (105) | 1.07 | 328  (71) | 351  (38) | 1.07 | 1.96  (0.21) | 1.89  (0.29) | 0.96 | 30.08 |
|  |  |  | 3000  inf | 940  (126) | 1027  (159) | 1.09 | 440  (82) | 526  (57) | 1.20 | 1.89  (0.30) | 1.89  (0.29) | 1.00 | 32.70 |
|  |  | 24-31 ^[11]^ | 1000  iv.bolus | 330  (18) | 346  (52) | 1.05 | NA |  |  | NA |  |  | 15.44 |
| **Vancomycin** | Caucasian | 25-30 ^[12]^ | 23.4 mg /kg  inf | 259.7 | 292.27 | 1.13 | NA | | | NA |  |  | 41.76 |
|  |  | 34-40 ^[13]^ | 500  inf | 98.03 | 90.69 | 0.93 | NA | | | NA |  |  | 23.37 |
|  | Chinese | 23±3 ^[14]^ | 1000  inf | 192.78  (20.2) | 139.12  (27.56) | 0.72 | 46.31  (4.48) | 30.65  (2.78) | 0.66 | 4.75  (0.59) | 5.89  (1.04) | 1.24 | 11.26 |
| **Cefuroxime** | Caucasian | 19-57 ^[15]^ | 250 | 32.6 | 25.0 | 0.77 | 46.7 | 40.9 | 0.88 | NA |  |  | 18.08 |
|  |  |  | 500 | 50.4 | 50.0 | 0.99 | 82.7 | 81.7 | 0.99 | NA |  |  | 22.55 |
|  |  |  | 1000 | 90.8 | 100.0 | 1.10 | 181.4 | 163.5 | 0.90 | NA |  |  | 104.3 |

AUC_inf_ area under the plasma concentration–time curve extrapolated to infinity; C_max_ maximum (peak) concentration of drug in blood plasma; CLr renal clearance; Obs observed value; Pre predicted value; P/O ratio of predicted value versus observed value;The mean absolute prediction error (MAPE) was additionally applied to examine and compare the precision and bias of simulations in PBPK models, as estimated using Eqs. 1: $\mathrm{MAPE}\left( \boldsymbol{\%} \right)\mathbf{=}\frac{\boldsymbol{1}}{\boldsymbol{n}}\boldsymbol{\times}\sum_{\boldsymbol{i}}^{\boldsymbol{n}} \frac{\left| \boldsymbol{(}\boldsymbol{C}_{\boldsymbol{Pred}\boldsymbol{,}\boldsymbol{i}}\boldsymbol{-}\boldsymbol{C}_{\boldsymbol{Obs}\boldsymbol{,}\boldsymbol{i}}\boldsymbol{)} \right|\boldsymbol{\times}\boldsymbol{10}\boldsymbol{0}}{\boldsymbol{C}_{\boldsymbol{Obs}\boldsymbol{,}\boldsymbol{i}}}$ where $C_{Pred,i}$ and $C_{Obs,i}$ denote the $i$th predicted and observed value, respectively.

| [1] (Harding and Harper 1983) | [2] (Drusano et al. 1984) | [3] (Harding et al. 1981) |
| --- | --- | --- |
| [4] (Bulitta et al. 2010) | [5] (Kalman et al. 1992) | [6] (Ljungberg and Nilsson-Ehle 1984) |
| [7] (Guangquan. et al. 1994) | [8] (Bryskier et al. 1990) | [9] (Bryskier et al. 1990) |
| [10] (Lenfant et al. 1995) | [11] (Korting et al. 1987) | [12] (Blouin et al. 1982) |
| [13] (Krogstad et al. 1980) | [14] (Jinyu et al. 2003) | [15] (Foord 1976) |

**Supplemental Table S4 Predicted versus observed PK parameters for Caucasian subjects with renal impairment**

| Drug | Age  (years) | Dose (mg)  Route | **GFR 60-90 ml/min** | | | | | | | | | | **GFR 30-60 ml/min** | | |
| --- | --- | --- | --- | --- | --- | --- | --- | --- | --- | --- | --- | --- | --- | --- | --- |
|  |  |  | AUC_inf_ (mg·h /L) | | | C_max_ (mg/L) | | | CLr (L/h) | | | MAPE  (%) | AUCinf (mg·h /L) | | |
|  |  |  | Obs | Pre | P/O | Obs | Pre | P/O | Obs | Pre | P/O |  | Obs | Pre | P/O |
| Ceftazidime | 26-74 ^[1]^ | 15 mg/kg  iv.bolus | 295.7 | 222.4 | 0.75 | 167 | 194 | 1.16 | 2.35 | 3.32 | 1.41 | 24.79 | 342.60 | 343.65 | 1.00 |
|  | 34-88 ^[2]^ | 1000  iv.bolus | 190.1 | 181.7 | 0.96 | NA |  |  | 4.82 | 3.11 | 0.65 | 4.41 | 285.71 | 338.42 | 1.18 |
|  | 27-91 ^[3]^ | 1000  iv.bolus | NA |  |  | NA |  |  | NA |  |  | NA | 335.84 | 312.96 | 0.93 |
| Cefodizime | 26-70 ^[4]^ | 1000  iv.bolus | NA |  |  | NA |  |  | NA |  |  | NA | 523.4  (27.8) | 592.3  (117.3) | 1.13 |
|  | 19-62 ^[5]^ | 1000  iv.bolus | NA |  |  | NA |  |  | NA |  |  | NA | 526.0 | 562.6 | 1.07 |
| Vancomycin | 53-67 ^[6]^ | 15 mg/kg  iv infusion | 221.9 | 263.6 | 1.19 | NA |  |  | NA |  |  | 35.20 | 314.0 | 451 | 1.43 |
|  | 18-85 ^[7]^ | Various  iv infusion | 310.58 | 300 | 0.97 | NA |  |  | NA |  |  | 15.14 | 740.71 | 644.91 | 0.87 |
|  | 35-87 ^[8]^ | 11.6/10.0  mg/kg  iv infusion | NA |  |  | NA |  |  | NA |  |  | NA | 264  (89) | 333  (55) | 1.26 |
| Cefuroxime | 26-55 ^[9]^ | 1000 mg  iv infusion | NA |  |  | NA |  |  | NA |  |  | NA | 175.7 | 281.68 | 1.60 |

**Supplemental Table S4 Predicted versus observed PK parameters for Caucasian subjects with renal impairment (to be continued)**

| Drug | **GFR 30-60 ml/min** | | | | | | | **GFR <30 ml/min** | | | | | | | | | |
| --- | --- | --- | --- | --- | --- | --- | --- | --- | --- | --- | --- | --- | --- | --- | --- | --- | --- |
|  | C_max_ (mg/L) | | | CLr(L/h) | | | MAPE  (%) | AUC_inf_ (mg·h /L) | | | C_max_ (mg/L) | | | CLr(L/h) | | | MAPE  (%) |
|  | Obs | Pre | P/O | Obs | Pre | P/O |  | Obs | Pre | P/O | Obs | Pre | P/O | Obs | Pre | P/O |  |
| Ceftazidime | 143.5  (67.3) | 188.33  (22.7) | 1.31 | 1.70  (0.25) | 2.01  (0.31) | 1.18 | 27.98 | 772.65  (119.4) | 480.19  (126.5) | 0.62 | 134.0 | 176.1 | 1.31 | 0.70  (0.23) | 0.98  (0.16) | 1.40 | 42.74 |
|  | NA |  |  | 2.66  (0.96) | 1.93  (0.33) | 0.73 | 26.39 | 763.36 | 517.55 | 0.68 | NA |  |  | 0.80  (0.29) | 0.95  (0.17) | 1.00 | 36.05 |
|  | NA |  |  | 2.40  (0.53) | 2.13  (0.26) | 0.89 | 45.76 | 581.9 | 540.72 | 0.93 | NA |  |  | 1.34  (0.51) | 0.97  (0.18) | 0.72 | 13.06 |
| Cefodizime | 220.6  (7.9) | 161.7  (25.37) | 0.73 | 1.04  (0.16) | 0.64  (0.09) | 0.62 | 41.03 | 1015.9  (127.0) | 743.1  (0.09) | 0.73 | 246.3  (11.7) | 157.8  (24.4) | 0.64 | 0.45  (0.12) | 0.31  (0.05) | 0.69 | 45.74 |
|  | 221 | 217 | 0.98 | NA |  |  | 35.72 | 628.08 | 686.07 | 1.09 | 274.7 | 195.9 | 0.71 | NA |  |  | 37.40 |
| Vancomycin | NA |  |  | NA |  |  | 43.63 | NA |  |  | NA |  |  | NA |  |  |  |
|  | NA |  |  | NA |  |  | 12.93 | 1595.0 | 1211.6 | 0.76 | NA |  |  | NA |  |  | 24.04 |
|  | NA |  |  | 2.89  (0.65) | 2.16  (0.30) | 0.75 | 26.14 | 451  (214) | 688  (142) | 1.53 | NA |  |  | 1.19  (0.47) | 0.98  (0.15) | 0.82 | 52.55 |
| Cefuroxime | NA |  |  | 3.82  (2.87) | 3.64  (0.26) | 0.95 | 60.32 | 1013.5 | 787.33 | 0.78 | NA |  |  | 1.26 | 1.95 | 1.55 | 22.32 |

AUC_inf_ area under the plasma concentration–time curve extrapolated to infinity; C_max_ maximum (peak) concentration of drug in blood plasma; CLr renal clearance; Obs observed value; Pre predicted value; P/O ratio of predicted value versus observed value.The mean absolute prediction error (MAPE) was additionally applied to examine and compare the precision and bias of simulations in PBPK models, as estimated using Eqs. 1: $\mathrm{MAPE}\left( \boldsymbol{\%} \right)\mathbf{=}\frac{\boldsymbol{1}}{\boldsymbol{n}}\boldsymbol{\times}\sum_{\boldsymbol{i}}^{\boldsymbol{n}} \frac{\left| \boldsymbol{(}\boldsymbol{C}_{\boldsymbol{Pred}\boldsymbol{,}\boldsymbol{i}}\boldsymbol{-}\boldsymbol{C}_{\boldsymbol{Obs}\boldsymbol{,}\boldsymbol{i}}\boldsymbol{)} \right|\boldsymbol{\times}\boldsymbol{100}}{\boldsymbol{C}_{\boldsymbol{Obs}\boldsymbol{,}\boldsymbol{i}}}$ where $C_{Pred,i}$ and $C_{Obs,i}$ denote the $i$th predicted and observed value, respectively.

| [1] (Leroy et al. 1984) | [2] (van Dalen et al. 1986) | [3] (Welage et al. 1984) |
| --- | --- | --- |
| [4] (Loffreda et al. 1999) | [5] (el Guinaidy et al. 1991) | [6] (Hurst et al. 1990) |
| [7] (Matzke et al. 1984) | [8] (Rodvold et al. 1988) | [9] (van Dalen et al. 1979) |

**Supplemental Table S5 Observed and predicted PK parameters of four model drugs for Chinese patients with renal impairment**

| Drug | Dose (mg)  Route | **Mild renal impairment** | | | | **Moderate renal impairment** | | | | **Severe renal impairment** | | | | | | | | | |
| --- | --- | --- | --- | --- | --- | --- | --- | --- | --- | --- | --- | --- | --- | --- | --- | --- | --- | --- | --- |
|  |  | AUC_inf_ (mg h/L)  Mean (SD) | | | MAPE  (%) | AUC_inf_ (mg h/L)  Mean (SD) | | | MAPE  (%) | AUC_inf_ (mg h/L)  Mean (SD) | | | CL_r_ (L/h)  Mean (SD) | | | C_max_ (mg/L)  Mean (SD) | | | MAPE  (%) |
|  |  | Obs | Pre | P/O |  | Obs | Pre | P/O |  | Obs | Pre | P/O | Obs | Pre | P/O | Obs | Pre | P/O |  |
| Ceftazidime | 2000 ^[1]^  iv.bolus | NA |  |  |  | NA |  |  |  | 1326.4  (658) | 1076.91  (196) | 0.81 | NA |  |  | NA |  |  | 18.8 |
|  | 2000  Multiple^a^ | 410  (13) | 501  (58) | 1.22 | 20.9 | 990  (264) | 1130 | 1.14 | 5.9 | 990  (264) | 1130 | 1.14 | NA |  |  | NA |  |  | 5.9 |
| Cefodizime | 16.2mg/kg^[2]^  iv.bolus | NA |  |  |  | NA |  |  |  | 896.24  (115.76) | 702.37  (169.39) | 0.78 | 0.46 (0.15) | 0.30 (0.06) | 0.65 | NA |  |  | 21.6 |
| Vancomycin | 1000 ^[3]^  iv infusion | 378.79 | 232.46 | 0.61 | 38.6 | 367.64 | 444.43 | 1.21 | 20.9 | 751.88 | 980.14 | 1.30 | NA |  |  | NA |  |  | 30.3 |
| Cefuroxime | 1000/1500 ^[4]^  iv infusion | NA |  |  |  | NA |  |  |  | 798.65  (362.83) | 601.98  (102.78) | 0.75 | NA |  |  | 79.33  (10.75) | 74.23  (9.72) | 0.94 | 17.1 |

AUC_inf_ area under the plasma concentration–time curve extrapolated to infinity; C_max_ maximum (peak) concentration of drug in blood plasma; CLr renal clearance; Obs observed values; Pre predicted values; P/O ratio of predicted values versus observed values; The mean absolute prediction error (MAPE) was additionally applied to examine and compare the precision and bias of simulations in PBPK models, as estimated using Eqs. 1: $\mathrm{MAPE}\left( \boldsymbol{\%} \right)\mathbf{=}\frac{\boldsymbol{1}}{\boldsymbol{n}}\boldsymbol{\times}\sum_{\boldsymbol{i}}^{\boldsymbol{n}} \frac{\left| \boldsymbol{(}\boldsymbol{C}_{\boldsymbol{Pred}\boldsymbol{,}\boldsymbol{i}}\boldsymbol{-}\boldsymbol{C}_{\boldsymbol{Obs}\boldsymbol{,}\boldsymbol{i}}\boldsymbol{)} \right|\boldsymbol{\times}\boldsymbol{100}}{\boldsymbol{C}_{\boldsymbol{Obs}\boldsymbol{,}\boldsymbol{i}}}$ where $C_{Pred,i}$ and $C_{Obs,i}$ denote the $i$th predicted and observed value, respectively; ^a^ after the 7th dose.

| [1] (Lin et al. 1989) | [2] (Li et al. 2000) | [3] (Ji et al. 2018) | [4] (Cheng et al. 2010) |
| --- | --- | --- | --- |

**Supplemental Table S6 Comparison of prediction performance of different population models by simulating the same clinical studies in Chinese renal impairment patients**

| **Drug** | **Age (yrs)** | **Dosing route** | **Level of renal impairment** | **MAPE (%)** | | |
| --- | --- | --- | --- | --- | --- | --- |
|  |  |  |  | **Chinese Healthy**  **volunteers Model** | **Caucasian renal impairment population model** | **Chinese renal impairment population model** |
| **Ceftazidime** | 21-75^[1]^ | 2000 mg.iv.bolus | Mild | 26.6 | 22.9 | 20.9 |
|  |  | 2000 mg.Multiple. iv.bolus | Moderate & Severe | 43.8 | 6.0 | 5.9 |
|  |  | 2000 mg.iv.bolus | Severe | 76.8 | 20.8 | 18.8 |
| **Cefodizime** | —^[2]^ | 16.2mg/kg.iv.bolus | Severe | 65.1 | 11.6 | 21.6 |
| **Vancomycin** | 42-95^[3]^ | 16.2mg/kg.iv.bolus | Mild | 64.9 | 22.9 | 38.6 |
|  |  |  | Moderate | 63.8 | 24.4 | 20.9 |
|  |  |  | Severe | 83.8 | 34.2 | 30.3 |
| **Cefuroxime** | 34-55^[4]^ | 1000/1500.iv.infusion | Severe | 86.5 | 38.4 | 17.1 |

The mean absolute prediction error (MAPE) was additionally applied to examine and compare the precision and bias of simulations in PBPK models, as estimated using Eqs. 1: $\mathrm{MAPE}\left( \boldsymbol{\%} \right)\mathbf{=}\frac{\boldsymbol{1}}{\boldsymbol{n}}\boldsymbol{\times}\sum_{\boldsymbol{i}}^{\boldsymbol{n}} \frac{\left| \boldsymbol{(}\boldsymbol{C}_{\boldsymbol{Pred}\boldsymbol{,}\boldsymbol{i}}\boldsymbol{-}\boldsymbol{C}_{\boldsymbol{Obs}\boldsymbol{,}\boldsymbol{i}}\boldsymbol{)} \right|\boldsymbol{\times}\boldsymbol{100}}{\boldsymbol{C}_{\boldsymbol{Obs}\boldsymbol{,}\boldsymbol{i}}}$ where $C_{Pred,i}$ and $C_{Obs,i}$ denote the $i$th predicted and observed value, respectively.

| [1] (Lin et al. 1989) | [2] (Li et al. 2000) | [3] (Ji et al. 2018) | [4] (Cheng et al. 2010) |
| --- | --- | --- | --- |

**Reference**

US FOOD & DRUG ADMINISTRATION (2015). "<CEFTAZIDIME for injection USP and DEXTROSE injection USP, for intravenous use.pdf>."Retrieved 05/21, 2020, from [https://www.accessdata.fda.gov/drugsatfda_docs/label/ 2015/206494s000lbl.pdf](https://www.accessdata.fda.gov/drugsatfda_docs/label/2015/206494s000lbl.pdf).

Abduljalil, K., X. Pan, A. Pansari, M. Jamei and T. N. Johnson (2019). "Preterm Physiologically Based Pharmacokinetic Model. Part II: Applications of the Model to Predict Drug Pharmacokinetics in the Preterm Population." Clin Pharmacokinet <http://doi.org/10.1007/s40262-019-00827-4>

Blouin, R. A., L. A. Bauer, D. D. Miller, K. E. Record and W. O. Griffen, Jr. (1982). "Vancomycin pharmacokinetics in normal and morbidly obese subjects." Antimicrob Agents Chemother **21**(4): 575-580 <http://doi.org/10.1128/aac.21.4.575>

Bryskier, A., T. Procyk, D. Tremblay, B. Lenfant and J. B. Fourtillan (1990). "Pharmacokinetics of cefodizime administered intravenously as a single-dose (1.0 and 2.0 g) to healthy adult volunteers." J Antimicrob Chemother **26 Suppl C**: 65-70 <http://doi.org/10.1093/jac/26.suppl_c.65>

Bryskier, A., T. Procyk, D. Tremblay, B. Lenfant and J. B. Fourtillan (1990). "The pharmacokinetics of cefodizime following intravenous and intramuscular administration of a single dose of 1.0 g." J Antimicrob Chemother **26 Suppl C**: 59-63 <http://doi.org/10.1093/jac/26.suppl_c.59>

Bulitta, J. B., C. B. Landersdorfer, S. J. Huttner, G. L. Drusano, M. Kinzig, U. Holzgrabe, . . . F. Sorgel (2010). "Population pharmacokinetic comparison and pharmacodynamic breakpoints of ceftazidime in cystic fibrosis patients and healthy volunteers." Antimicrob Agents Chemother **54**(3): 1275-1282 <http://doi.org/10.1128/AAC.00936-09>

Cheng, W., D. Li, X. Liu, G. Liu, Y. lv and A. Shan (2010). "Pharmacokinetics of single intravenous cefuroxime in patients with renal insufficiency." The Chinese Journal of Clinical Pharmacology **26**(05): 339-343 <http://doi.org/10.13699/j.cnki.1001-6821.2010.05.013>

Dallmann, A., I. Ince, J. Solodenko, M. Meyer, S. Willmann, T. Eissing and G. Hempel (2017). "Physiologically Based Pharmacokinetic Modeling of Renally Cleared Drugs in Pregnant Women." Clin Pharmacokinet **56**(12): 1525-1541 <http://doi.org/10.1007/s40262-017-0538-0>

Drusano, G. L., H. C. Standiford, B. Fitzpatrick, J. Leslie, P. Tangtatsawasdi, P. Ryan, . . . S. C. Schimpff (1984). "Comparison of the pharmacokinetics of ceftazidime and moxalactam and their microbiological correlates in volunteers." Antimicrob Agents Chemother **26**(3): 388-393 <http://doi.org/10.1128/aac.26.3.388>

el Guinaidy, M. A., S. Nawishy, M. Abd el Bary and M. S. Sabbour (1991). "Pharmacokinetics of cefodizime in normal individuals and in patients with renal failure." Chemotherapy **37**(2): 77-85 <http://doi.org/10.1159/000238837>

Foord, R. D. (1976). "Cefuroxime: human pharmacokinetics." Antimicrob Agents Chemother **9**(5): 741-747 <http://doi.org/10.1128/aac.9.5.741>

Guangquan., Z., X. Guangxia., Z. Yaping., D. Hui. and Z. Guandong. (1994). "Pharmacokinetic changes in burned patients treated with Ceftazidime." Chinese Journal of Plastic Surgery (05): 385-388

Harding, S. M. and P. B. Harper (1983). "The pharmacokinetic behaviour of ceftazidime in man and the relationship between serum levels and the in vitro susceptibility of clinical isolates." Infection **11 Suppl 1**: S49-53 <http://doi.org/10.1007/BF01641107>

Harding, S. M., A. J. Monro, J. E. Thornton, J. Ayrton and M. I. Hogg (1981). "The comparative pharmacokinetics of ceftazidime and cefotaxime in healthy volunteers." J Antimicrob Chemother **8 Suppl B**: 263-272 <http://doi.org/10.1093/jac/8.suppl_b.263>

Hurst, A. K., M. A. Yoshinaga, G. H. Mitani, K. A. Foo, R. W. Jelliffe and E. C. Harrison (1990). "Application of a Bayesian method to monitor and adjust vancomycin dosage regimens." Antimicrob Agents Chemother **34**(6): 1165-1171 <http://doi.org/10.1128/aac.34.6.1165>

Ji, X. W., S. M. Ji, X. R. He, X. Zhu, R. Chen and W. Lu (2018). "Influences of renal function descriptors on population pharmacokinetic modeling of vancomycin in Chinese adult patients." Acta Pharmacol Sin **39**(2): 286-293 <http://doi.org/10.1038/aps.2017.57>

Jinyu, H., S. Yaoguo, Z. Jin, Y. Jicheng, Z. Yinyuan and C. Guoying (2003). "Pharmacokinetics of vancomycin in elderly and young healthy volunteers." Chin J Infect Chemother **3**(03): 138-142 <http://doi.org/10.16718/j.1009-7708.2003.03.004>

Kalman, D., S. L. Barriere and B. L. Johnson, Jr. (1992). "Pharmacokinetic disposition and bactericidal activities of cefepime, ceftazidime, and cefoperazone in serum and blister fluid." Antimicrob Agents Chemother **36**(2): 453-457 <http://doi.org/10.1128/aac.36.2.453>

Korting, H. C., M. Schafer-Korting, L. Maass, N. Klesel and E. Mutschler (1987). "Cefodizime in serum and skin blister fluid after single intravenous and intramuscular doses in healthy volunteers." Antimicrob Agents Chemother **31**(11): 1822-1825 <http://doi.org/10.1128/aac.31.11.1822>

Krogstad, D. J., R. C. Moellering, Jr. and D. J. Greenblatt (1980). "Single-dose kinetics of intravenous vancomycin." J Clin Pharmacol **20**(4): 197-201 <http://doi.org/10.1002/j.1552-4604.1980.tb01696.x>

Lenfant, B., F. Namour, C. Logeais, D. Coussediere, O. Rivault, A. Bryskier and A. Surjus (1995). "Pharmacokinetics of cefodizime following single doses of 0.5, 1.0, 2.0, and 3.0 grams administered intravenously to healthy volunteers." Antimicrob Agents Chemother **39**(9): 2037-2041 <http://doi.org/10.1128/aac.39.9.2037>

Leroy, A., F. Leguy, F. Borsa, G. R. Spencer, J. P. Fillastre and G. Humbert (1984). "Pharmacokinetics of ceftazidime in normal and uremic subjects." Antimicrob Agents Chemother **25**(5): 638-642 <http://doi.org/10.1128/aac.25.5.638>

Li, R., R. Song and W. Hu (2000). "Pharmacokinetic study and clinical application of cefodizime in patients with different renal impairment." Chinese Journal of Antibiotics(02): 117-119 <http://doi.org/10.13461/j.cnki.cja.002725>

Lin, M. S., L. S. Wang and J. D. Huang (1989). "Single- and multiple-dose pharmacokinetics of ceftazidime in infected patients with varying degrees of renal function." J Clin Pharmacol **29**(4): 331-337 <http://doi.org/10.1002/j.1552-4604.1989.tb03337.x>

LinMS., W. LS. and H. JD. "Single- and multiple-dose pharmacokinetics of ceftazidime in infected patients with varying degrees of renal function." J Clin Pharmacol: 331-337 <http://doi.org/10.1002/j.1552-4604.1989.tb03337.x>

Ljungberg, B. and I. Nilsson-Ehle (1984). "Pharmacokinetics of ceftazidime in elderly patients and young volunteers." Scand J Infect Dis **16**(3): 325-326 <http://doi.org/10.3109/00365548409070410>

Loffreda, A., E. Lampa, C. Lucarelli, M. Amorena, C. Contaldi, V. Calderaro and F. Rossi (1999). "Pharmacokinetics of cefodizime in patients with various degrees of renal failure." Chemotherapy **45**(1): 1-7 <http://doi.org/10.1159/000007158>

Matzke, G. R., R. W. McGory, C. E. Halstenson and W. F. Keane (1984). "Pharmacokinetics of vancomycin in patients with various degrees of renal function." Antimicrob Agents Chemother **25**(4): 433-437 <http://doi.org/10.1128/aac.25.4.433>

Rodvold, K. A., R. A. Blum, J. H. Fischer, H. Z. Zokufa, J. C. Rotschafer, K. B. Crossley and L. J. Riff (1988). "Vancomycin pharmacokinetics in patients with various degrees of renal function." Antimicrob Agents Chemother **32**(6): 848-852 <http://doi.org/10.1128/aac.32.6.848>

Schwameis, R., S. Syre, D. Marhofer, A. Appelt, D. Burau, K. Sarahrudi, . . . M. Zeitlinger (2017). "Pharmacokinetics of Cefuroxime in Synovial Fluid." Antimicrob Agents Chemother **61**(10) <http://doi.org/10.1128/AAC.00992-17>

van Dalen, R., T. B. Vree, A. M. Baars and E. Termond (1986). "Dosage adjustment for ceftazidime in patients with impaired renal function." Eur J Clin Pharmacol **30**(5): 597-605 <http://doi.org/10.1007/BF00542421>

van Dalen, R., T. B. Vree, J. C. Hafkenscheid and J. S. Gimbrere (1979). "Determination of plasma and renal clearance of cefuroxime and its pharmacokinetics in renal insufficiency." J Antimicrob Chemother **5**(3): 281-292 <http://doi.org/10.1093/jac/5.3.281>

Welage, L. S., R. W. Schultz and J. J. Schentag (1984). "Pharmacokinetics of ceftazidime in patients with renal insufficiency." Antimicrob Agents Chemother **25**(2): 201-204 <http://doi.org/10.1128/aac.25.2.201>
